# A New Method for Growth Factor Enrichment from Dairy Products by Electrodialysis with Filtration Membranes: The Major Impact of Raw Product Pretreatment

**DOI:** 10.3390/ijms25137211

**Published:** 2024-06-29

**Authors:** Sabita Kadel, Vladlen Nichka, Jacinthe Thibodeau, Behnaz Razi Parjikolaei, Laurent Bazinet

**Affiliations:** 1 Dairy Science and Technology Research Center (STELA), Institute of Nutrition and Functional Foods (INAF), Department of Food Sciences, Laval University, Quebec, QC G1V 0A6, Canada; sabita.kadel.1@ulaval.ca (S.K.); vladlen.nichka.1@ulaval.ca (V.N.), jacinthe.thibodeau.1@ulaval.ca (J.T.); 2 Laboratory of Food Processing and ElectroMembrane Processes (LTAPEM), Laval University, Quebec, QC G1V 0A6, Canada; 3 Arla Food Ingredients, Soenderupvej 26, DK-6920 Videbæk, Denmark; bepar@arlafoods.com

**Keywords:** filtration membrane, electrodialysis with filtration membranes, growth factor, separation, colostrum, whey, pretreatment

## Abstract

This study is focused on fractionation of insulin-like growth factor I (IGF-I) and transforming growth factor-β2 (TGF-β2) using a new electro-based membrane process calledelectrodialysis with filtration membranes (EDFM). Before EDFM, different pretreatments were tested, and four pH conditions (4.25, 3.85, 3.45, and 3.05) were used during EDFM. It was demonstrated that a 1:1 dilution of defatted colostrum with deionized water to decrease mineral content followed by the preconcentration of GFs by UF is necessary and allow for these compounds to migrate to the recovery compartment during EDFM. MS analyses confirmed the migration, in low quantity, of only α-lactalbumin (α-la) and β-lactoglobulin (β-lg) from serocolostrum to the recovery compartment during EDFM. Consequently, the ratio of GFs to total protein in recovery compartment compared to that of feed serocolostrum solution was 60× higher at pH value 3.05, the optimal pH favoring the migration of IGF-I and TGF-β2. Finally, these optimal conditions were tested on acid whey to also demonstrate the feasibility of the proposed process on one of the main by-products of the cheese industry; the ratio of GFs to total protein was 2.7× higher in recovery compartment than in feed acid whey solution, and only α-la migrated. The technology of GF enrichment for different dairy solutions by combining ultrafiltration and electrodialysis technologies was proposed for the first time.

## 1. Introduction

Growth factors (GFs), which are soluble proteins or polypeptides having the primary function of cellular proliferation and/or differentiation, are abundantly present in milk and dairy products [1]. An increasing number of studies on milk-derived GFs have demonstrated their high potential as bioactive ingredients in a range of functional foods and have proposed several health-related applications, the main being the treatment of skin disorders, such as psoriasis; the gut; and bone health [2,3,4]. They have already been used to develop therapeutic compositions for wound healing and the treatment of gastrointestinal diseases [3,4,5,6]. Indeed, GFs have been shown to be an effective oral treatment in children with active Crohn’s disease [3,4].

The most abundant GFs in milk and dairy solutions are insulin-like growth factor (IGF-I), transforming growth factor-β2 (TGF-β2), epidermal growth factor (EGF), and basic fibroblast growth factor (bFGF or FGF-2) [1,7]. Their fractionation is challenged by several factors, such as the range of their molecular weights and isoelectric points relative to that of other constituents in milk or dairy solutions and the fact that most of them are bonded or associated with other constituents [4]. In addition to this, their concentrations in dairy fluids are very low: less than 0.001 g/L in milk and less than 0.004 g/L in colostrum [1,8]. However, their concentrations needed for activity are in the order of micrograms or even nanograms per liter [8]. Several technological approaches have been developed to produce GF-enriched fractions from milk, colostrum, or whey, which can be classified into two main categories [4]: charge-based chromatographic processes [9,10,11] and size-based membrane separations [7,12,13]. Nevertheless, both methods have some disadvantages: chromatography is relatively expensive considering the high selectivity achieved, and membrane technology is associated with low selectivity and fouling due to the accumulation of opposite-charged molecules on the membrane surface.

In recent years, the use of an electrically driven process, such as electrodialysis in combination with filtration membranes (known as electrodialysis with filtration membranes (EDFM)), has been introduced and developed to increase the selectivity of molecules according to their size and charge [14]. The main principle of EDFM is that charged molecules migrate towards the anode or cathode under an electric field and are separated based on their charge and molecular weight (due to the main parameter of filtration membrane–molecular weight cut-off (MWCO)). Thus, this technology combines size- and charge-exclusion mechanisms to achieve the maximal process efficiency while reducing membrane fouling in comparison with conventional baromembrane processes. To the best of our knowledge, this technology has never been used for GF fractionation before, but it has already been used successfully for the separation of small, charged molecules, such as peptides, from various sources [14,15,16,17] and macromolecules, such as lactoferrin (LF) and bovine serum albumin (BSA), from whey or model solutions [18,19,20]. EDFM and electrodialysis (ED) processes in general are promising technologies for valorizing liquid waste from the food industry to extract valuable compounds, such as proteins and peptides, to create a circular economy and, in the long term, an eco-circular economy [21]. It is worth noting that ED technologies are not only environmentally friendly but also economically beneficial in comparison with traditional approaches to produce the GF-enriched fractions mentioned earlier [21].

In this context, the goal of this study was to demonstrate for the first time, the feasibility of EDFM technology to produce GF-enriched fractions (with IGF-I and TGF-β2) from dairy solutions. To achieve this goal, the following specific objectives were proposed: (1) to find the best pretreatment conditions for reasonable diminuation of mineral content and concentration of GFs in the feed product to be treated by EDFM; (2) to study the impact of pH on GFs and the protein migration rate during EDFM; (3) to evaluate EDFM performances in terms of relative energy consumption during GF enrichment; and (4) to demonstrate the efficiency of the developed process on different dairy products.

## 2. Results and Discussion

### 2.1. Pretreatment of Colostrum

Based on the pretest results, in the case of using 10% acetic acid (drop-by-drop addition to lower the pH to 3.8) without diluting defatted colostrum, the separation of caseins from other soluble proteins was noted. However, the analysis of MS chromatograms suggested that there were still soluble caseins present in the serocolostrum. Furthermore, another problem was also noticed on the serocolostrum: it formed a gel upon defrosting after being frozen at −20 °C.

During another pretest, a dilution ratio of 6:25 was tested prior to acidification with 10% acetic acid until it reached pH 4.6 (isoelectric point of caseins). This ratio was chosen based on the established protocol for casein precipitation in milk by acetic acid. Caseins were well separated, and serocolostrum was successfully prepared after this trial. But the serocolostrum was too diluted, and for both aforementioned pretreatment conditions, there was a problem of a strong pungent odor of acetic acid.

Thereby, another approach with the use of 1M HCl until pH 3.8 was tested for casein removal. Thus, at first, HCl was used without any dilution of serocolostrum. However, after centrifugation, it was noticed that there was no separation of caseins from other soluble proteins. Indeed, the electrical conductivity of defatted colostrum was high (about 5.2 mS/cm) because of the presence of four and five times more calcium and phosphorous, respectively, and five times more proteins than in the milk [22]. High concentration of minerals in defatted colostrum may have resulted in significant salting-in during HCl acidification, which tends to slow down the formation of protein aggregates and increase their solubility, leading to slower protein precipitation.

Therefore, in the last trial, first, defatted colostrum was diluted with deionized water in the ratio of 1:1 to lower the concentration of minerals and, hence, the salting-in effect during HCl acidification. Then, 1 M HCl was added drop by drop to 1:1 diluted defatted colostrum while vortexing until pH lower to 3.8. After centrifugation (11,000× *g*, 20 min at 4 °C), caseins were well separated, and serocolostrum was successfully prepared under these conditions. Thus, the acidification of 1:1 diluted defatted colostrum using 1 M HCl was found to be the best condition for removal of caseins and was therefore chosen to prepare serocolostrum for the EDFM experiments. It is worth noting that the dilution step is critical for the proposed process since otherwise it is impossible to obtain a suitable product for the GF enrichment process.

### 2.2. Separation of GFs from Concentrated Serocolostrum by EDFM

#### 2.2.1. Total Protein Concentration and Migration Rate

**Figure 1:**
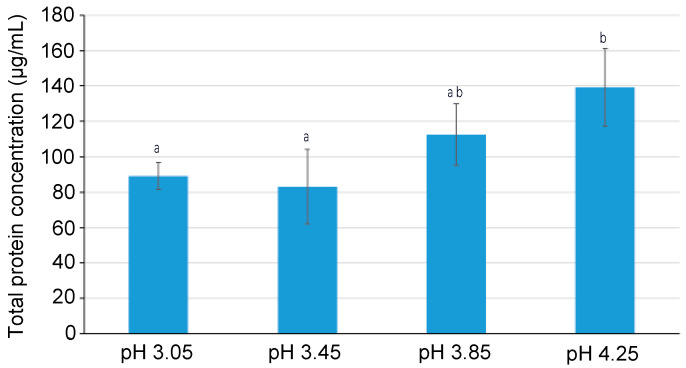
Total protein concentration in recovery compartment after 180 min of EDFM experiment for different pH conditions. Data with different letters are significantly different.

Total protein concentration in the recovery compartment after 180 min of the EDFM experiment at the four different pH conditions tested are presented in Figure 1. The result showed that the total protein migration from the feed to the recovery compartment decreased with a decrease in pH value. Consequently, the highest protein concentration was noticed in the recovery compartment at the highest pH condition tested, i.e., at pH 4.25, with a final value of 139 ± 22 μg/mL corresponding to a migration rate of 2.99 ± 0.62 g/m^2^·h, which was significantly higher than at pH 3.45 and pH 3.05 (*p* = 0.011). No significant difference in protein migration/protein concentration was observed at pH 3.85 and pH 4.25 due to large standard deviations. Similarly, the final protein concentration was statistically the same for pH conditions 3.85, 3.45, and 3.05. Compared to pH 4.25, the protein migration rate was 26% lower with the value of 2.20 ± 0.43 g/m^2^·h for pH 3.85 and 50% lower with the average value of 1.47 ± 0.18 g/m^2^·h for pH conditions 3.45 and 3.05. For all the pH conditions tested, there was a linear increase in protein concentration as a function of time during the EDFM process.

In the present study, since it was the first time that the GF separation by EDFM from serocolostrum or other products was tested, no information is available in the literature for these specific molecules. However, the fact that the pH impacts their migration is in adequation with data already reported in the literature for peptides [14,15] and proteins [18]. Indeed, the charge of proteins/peptides primarily depends on the pH of the solution, and their migration to respective recovery compartment depends on their charges at the working pH. Since the pI values of IGF-I and TGF-b2 were, respectively, 7.8–8.5 [4] and 7.7 [3,4], they had a positive charge at the working pH range between 4.25 and 3.05. Furthermore, their positive charge increased as the pH decreased, facilitating their migration towards the cathode. Hence, in the pioneering study of Poulin et al. [14], on peptide migration in an EDFM system, it was found that the total migration of cationic peptides from feed (β-lg hydrolysate) to the cationic recovery compartment decreased with an increase in hydrolysate solution pH while the opposite occurred for the anionic recovery compartment. Similar trends of decreased cationic peptide migration to the cationic recovery compartment from feed with an increase in the pH of the solution was noticed by Firdaous et al. [15] from an alfalfa white protein concentrate hydrolysate and more recently by Durand et al. [23] from a herring milt hydrolysate. Concerning lactoferrin (LF), Ndiaye et al. [18] reported that LF migration rates at the end of the treatments were 14.6%, 5.7%, and 4.4%, respectively at pH 3.0, pH 4.0, and pH 5.0.

Concerning the total migration rate obtained in this study, it was in good agreement with what was previously observed in the different studies already published and work on proteins/peptides: for cationic peptides, migration rates ranged from 0.097 g/m^2^·h [24] to 18.7 g/m^2^·h [25], while for positively charged proteins, they ranged between 0.4 g/m^2^·h [19] and 8.9 g/m^2^·h [18] for LF, between 5.3 g/m^2^·h [18] and 41 g/m^2^·h [19] for β-lg, and between 7.4 g/m^2^·h [19] and 29.1 g/m^2^·h [18] for BSA in model whey or whey-enriched solutions.

#### 2.2.2. Protein Characterization and Quantification

**Figure 2:**
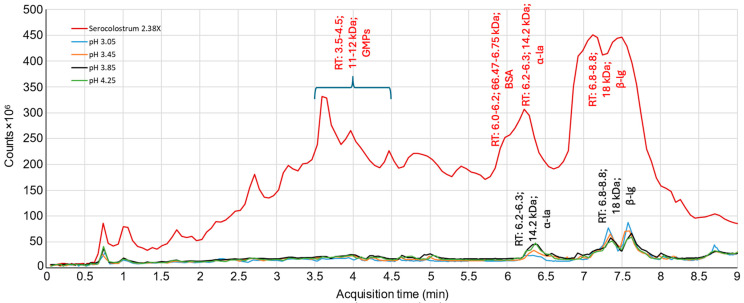
MS chromatogram of serocolostrum 2.38× in feed (T0) and cationic fractions (recovered after 180 min of EDFM experiment).

The major proteins present in the feed (serocolostrum) and the cationic recovery compartment were identified and quantified (relative abundances of protein) by RP-UPLC-MS-QTOF, and the MS chromatograms are presented in Figure 2. Regardless of the pH conditions used, out of four major proteins (GMPs, BSA, α-la, and β-lg) identified in the feed, only α-la and β-lg were migrated to the recovery compartment, except for pH 3.05, for which there was a migration of only β-lg. Furthermore, some trends appeared from the chromatograms, such as that the migration of α-la would decrease with a decrease in pH, while the opposite was observed for β-lg, which would increase with a decrease in pH.

Concerning β-lg, this increase in migration to the recovery compartment with a decrease in pH value was not statistically significant (*p* = 0.338), but a trend is highlighted; its highest migration to the recovery compartment was noticed at pH 3.05, which represented 2.32 ± 0.1% of its initial concentration in the feed solution, followed by pH 3.45 (1.78 ± 0.88%), pH 3.85 (1.67 ± 0.10%), and pH 4.25 (1.63 ± 0.14%). This could be explained, first, by the fact that β-lg exists as a monomer (≈18 kDa) at pH < 3.5, while it exists as an octamer (MW ≈ 144 kDa) in the pH range between 3.5 and 5.5 [26]. In addition, the charge of β-lg (pI = 5.2 [27], which is positive under its pI, increases as the pH decreases, as does its electrophoretic mobility, thereby allowing for a better migration of this protein to the recovery compartment in association with its decreasing size. Indeed, for LF migration, Ndiaye et al. [18] observed that the positive electrophoretic mobility of the LF in water decreased progressively from 3.0 × 10^−8^ m^2^·V^−1^·s^−1^ to 0.5 × 10^−8^ m^2^·V^−1^·s^−1^ while increasing the pH from 3.0 to 7.2, and that resulted in a higher migration of LF during EDUF treatment at pH 3.

Therefore, in this study, for the pH conditions lower than 3.5, in addition to the higher electrophoretic mobility of β-lg, there must have been lower/insignificant steric hindrance from the hydration layer or friction in the membrane pores for the negatively charged β-lg monomer compared to that of the octamer (less negatively charged), thereby facilitating its migration through the 300 kDa membrane [28,29]. In addition, its decreased global net charge as it approaches its pI value and, therefore, its decreasing electrophoretic mobility must have slowed down its migration [18]. Ndiaye et al. [18] also observed that the migration of β-lg during EDFM treatment from an enriched whey solution was optimal at pH 3.0 in comparison with pH values of 4.0 and 5.0. On the other hand, for the pH conditions >3.5, the β-lg octamer must have experienced a significant steric restriction to migrate through the membrane due to its hydrated radius and/or may have accumulated at the interface of the membrane due to their more complex conformation in accordance with the presence of eight monomers in the structure. Similar results of hindered migration have also been reported in the literature for charged chitosan oligomers (mixture of dimers, trimers, and tetramers) during EDFM, as their size increases and electrophoretic mobility decreases (due to their differences in molecular weight and charge) [30]. Indeed, previous studies on electromembrane-based processes have demonstrated that the MWCO of filtration membranes should be about 10 times higher than the size of the charged molecules for their successful transport through filtration membranes [30,31].

On the contrary, a decrease in α-la migration from the feed to the recovery compartment with a decrease in pH values was noticed, which represented 3.51 ± 0.81%, 3.49 ± 1.46%, and 2.07 ± 0.32% of its concentration in the feed solution for 4.25, 3.85, and 3.45, respectively. However, no significant difference was observed among the three pH values due to low migration and, consequently, large standard deviation (*p* = 0.199). Such an impact of pH on α-la migration during EDFM could be explained by its aggregation behavior at an acidic pH. Indeed, with a decrease in pH (more acidic), α-la undergoes significant structural changes upon the loss of bound Ca^2+^ from the binding pocket, resulting in a molten globule state. This leads to α-la aggregation, most probably due to a combination of hydrophobic and electrostatic interactions. Though the global net charge of the α-la aggregate increased with a decrease in pH value (pI of α-la = 4.5–4.8 [32,33]), an increase in size inversely affects its electrophoretic mobility and, therefore, its migration [18]. In addition, as explained in the previous paragraph, with an increase in the size/molecular weight of the charged molecules (the formation of aggregation), steric hindrance and/or friction in the membrane pores must have annihilated the migration of charged molecules through the membrane [30,34,35]. This result is in contradiction with the study of Ndiaye et al. [18], which reported that the migration of α-la as well as for β-lg, BSA, and lactoferrin was optimal at pH 3.0. This could be due to the difference in the membrane used and, consequently, the difference in physico-chemical properties (500 kDa PES UF membrane (thickness = 320 μm, zeta potential −17 mV, and electrical conductivity of 10.9 mS/cm [36]) vs. 300 kDa PES UF (thickness = 221 μm, zeta potential −11 mV and conductivity of 9.1 mS/cm) in the present study), such as the pore size and contact angle of these membranes, which are not mentioned in these studies, which can greatly impact the migration of charged species [28,29] or their adsorption/rejection at the membrane surface [19].

#### 2.2.3. Growth Factor Quantification, Recovery Yield, and Enrichment

**Figure 3:**
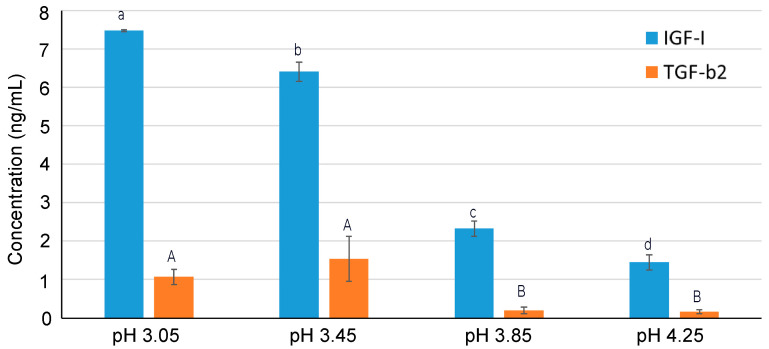
The concentration of IGF-I and TGF-β2 in the recovery compartment after 180 min of the EDFM experiment. Data with different letters are significantly different. Lowercase letters indicate differences for IGF-I concentration; uppercase—for TGF-β2 concentration.

The total concentration of the GFs of interest (IGF-I and TGF-β2) in the recovery compartment after 180 min of the EDFM experiment at the four different pH values tested were quantified by ELISA and are presented in Figure 3. As the pH value of the feed and recovery compartment decreases, the migration of IGF-I was found to be greatly enhanced (*p* ≤ 0.001). Therefore, its highest concentration in the recovery compartment was noticed at pH 3.05, corresponding to a value of 7.47 ± 0.03 ng/mL. This value represented 2.96 ± 0.12% of the IGF-I concentration present in the serocolostrum (before concentration by 5 kDa UF). As the pH values increased, the IGF-I concentration in the recovery compartment decreased and represented 2.53 ± 0.08%, 0.90 ± 0.06%, and 0.57 ± 0.06% of its initial concentration in the serocolostrum. Consequently, as expected, the recovery yield of IGF-I was the highest at pH 3.05 and the lowest at pH 4.25 (Table 1).

The migration trend of TGF-β2 was found to be more or less similar to that of IGF-I. As a result, its highest migration was observed at the lowest pH conditions, i.e., pH 3.05 and pH 3.45 (statistically insignificant between these two pH values; *p* = 0.452), corresponding to an averaged value of 1.31 ± 0.47 ng/mL, which was significantly higher (*p* ≤ 0.023) than at the pH conditions 3.85 and 4.25. This value represented 1.01 ± 0.33% of the TGF-β2 concentration present in the serocolostrum (before a concentration of 5 kDa UF). Its concentration at the pH values 3.85 and 4.25 were statistically the same (*p* = 0.996), which represented 0.16 ± 0.04% of its concentration in the initial serocolostrum. Consequently, as expected, the recovery yield of TGF-β2 was the highest at pH 3.05 and the lowest at pH 4.25 (Table 1).

The fact that the higher migration and, consequently, the higher recovery yield of IGF-I compared to TGF-β2 from the feed to the recovery compartments for any pH conditions is due to the higher concentrations of the former than the latter in the feed solution, as demonstrated by ELISA. Indeed, the concentration of IGF-I and TGF-β2 in the concentrated serocolostrum was 305.16 ± 2.34 ng/mL and 172.55 ± 2.41 ng/mL, respectively. Bargeman et al. [31] and Aider et al. [30] reported similar results of the increased transport rate of bioactive peptides and chitosan oligomers, respectively, with an increase in their concentration in the feed solution during electromembrane filtration.

The increased concentration of both GFs in the recovery compartment with a decrease in solution pH must be the result of the synergistic effect of various phenomena. The isoelectric point of IGF-I and TGF-β2 are 7.8–8.5 and 7.7, respectively. The pH conditions studied in this work were less than these two aforementioned pH values. Therefore, with a decrease in pH value, these proteins become more and more positively charged, contributing to their higher electrophoretic mobility and, consequently, their facilitated migration through the membrane from the feed to the recovery compartment. As mentioned previously, a similar phenomenon of the increased migration of lactoferrin with a decrease in pH due to its increased electrophoretic mobility was reported by Ndiaye et al. [18]. On the other hand, the PES 300 kDa filtration membrane used in this study is negatively charged at all the pH conditions studied [28,29]. Therefore, an increased migration of the GFs of interest with a decrease in pH could be explained by the electrostatic interactions (attraction) occurring between GFs and the membrane surface at the interface. Consequently, the migration of GFs must have been facilitated with a decrease in pH value due to the Donnan effect. A similar phenomenon was reported by Kadel et al., where the highest migration of a globally negatively charged bioactive peptide (from whey protein hydrolysate) was noticed when a positively charged PVDF 30 kDa filtration membrane was used [29].

**Figure 4:**
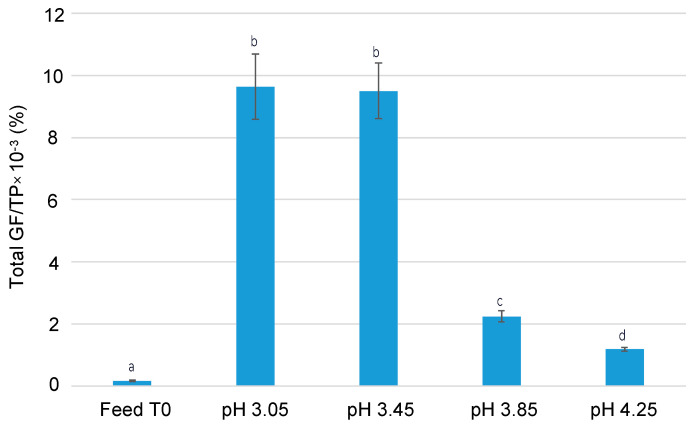
The enrichment of the GFs of interest (IGF-I+TGF-β2) in the recovery compartment at T180. Data with different letters are significantly different.

To determine if the recovered fractions, after 180 min of EDFM experiments, were enriched with GFs compared to fractions present in the feed solution, calculations were carried out using Equation (3), as explained in Section 3.5.3 and presented in Figure 4. Interestingly, regardless of the pH conditions during the EDFM experiment, the recovered fractions were significantly enriched with GFs compared to the fractions in the feed solution (*p* < 0.001). Furthermore, the ratio of GFs (IGF-I+TGF-β2) to total protein was the highest at pH values of 3.05 and 3.45 (60× higher in recovery than in the feed solution), which significantly decreased with an increase in pH values (*p* < 0.001). Consequently, the GF-to-total protein ratio at pH 3.85 and 4.25 was found to be ≈14× and 7× higher, respectively, in recovery compared to that in feed solution. As mentioned previously, there is no information available in the literature, as this is the first time that such a process is used to concentrate GFs. However, a pressure-driven process has been used previously to concentrate GFs in retentate. A study performed by Akbache et al. successfully concentrated TGF-β2 by a 13× factor using 10 kDa UF/DF from MF-Whey, but there was a loss of IGF-I (75–98%) (transmission through membrane to permeate) because of its lower molecular mass (7.5 kDa) [7].

#### 2.2.4. Relative Energy Consumptions

**Figure 5:**
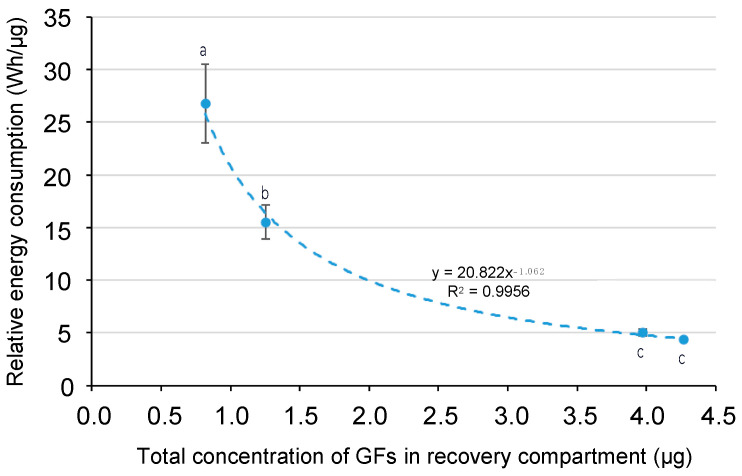
The evolution of relative energy consumption as a function of the total concentration of GFs in the recovery compartment after 180 min of EDFM. Data with different letters are significantly different.

The energy consumption that was required for the migration of 1 microgram (μg) of the GFs of interest (IGF-I+TGF-β2) to the recovery compartment was calculated using Equation (5), discussed in Section 3.5.4. It should be noted that under the constant potential difference of 9 V during EDFM, the current intensity was in the range of 1 A to 0.5 A approximately, with minor deviations depending on the pH conditions used. Relative energy consumption was found to be 4.33, 5.03, 15.52, and 26.78 Wh/μg for pH 3.05, 3.45, 3.85, and 4.25, respectively. These values demonstrated that the relative energy consumption decreased with a decrease in pH value and reached almost a plateau after pH 3.45 (Figure 5); as expected, when the migration of GFs was the highest, the energy consumption was the lowest and vice versa. More GFs migrated to the recovery compartment since their higher negative charges favored their migration and, consequently, the relative energy consumption required for their migration was lower. Except for pH 3.05 and 3.45, the relative energy consumption for GFs increased significantly with an increase in pH value (*p* ≤ 0.001). However, the relative energy consumption in the present study is high (between 4330 and 26,780 kWh/g) in comparison with data reported in the literature for proteins/peptides. The range in relative energy consumption reported in the literature for EDUF or, more generally, the EDFM process is between 0.0035 and 25 kWh/g [18,19,23,25,37]. Such a high energy consumption is mainly explained by the low concentration of GFs in the feed solution, and whatever their concentrations, they were increased by UF prior to EDFM. Indeed, the main part of the current is used to migrate ions present in the solution. Furthermore, if GFs were not concentrated in the feed solution, it would have been impossible to migrate and concentrate them.

### 2.3. Separation of GFs from Concentrated Acid Whey by EDFM

To confirm the feasibility of the production of GF-enriched fractions, the same pretreatment and EDFM process were investigated under the optimal conditions obtained previously on acid whey, a product generated in large volumes in the dairy industry [38].

**Figure 6:**
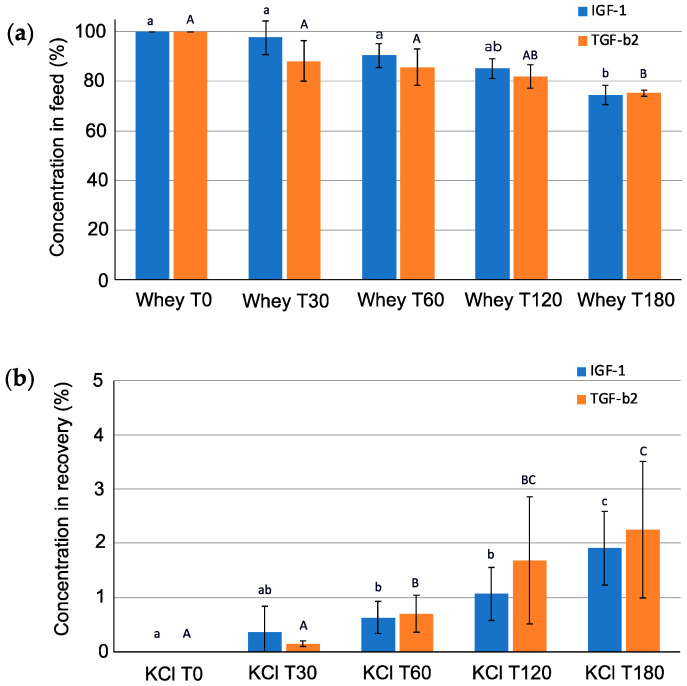
The concentration of TGF-β2 and IGF-I in the (**a**) feed and (**b**) recovery compartments during 180 min of EDFM of whey. Data with different letters are significantly different. Lowercase letters indicate differences for IGF-I concentration; uppercase—for TGF-β2 concentration.

The average concentration of the GFs of interest (TGF-β2 and IGF-I) in the feed and recovery compartment during the EDFM of the prepared acid whey (at T = 0, 30, 60, 120, and 180 min) was quantified by ELISA and presented on Figure 6. In the case of the feed compartment, the percentage of both GFs decreased (from 100%, corresponding to concentration of TGF-β2 of 12.51 ± 6.05 ng/mL and IGF-1 of 6.22 ± 0.18 ng/mL, to 75.16 ± 1.30% for TGF-β2, and to 74.46 ± 3.83% for IGF-I; *p* < 0.001), while in the recovery compartment, these percentages increased (from 0% to 2.25 ± 1.26% for TGF-β2 and to 1.91 ± 0.68% for IGF-I; *p* < 0.001). The recovery yield in the recovery compartment after 180 min of the EDFM of whey was also calculated using Equation (2), as described in Section 3.5.3. Thus, the recovery yield in case of TGF-β2 represented 1.90 ± 1.13%, while for IGF-I, it was equal to 1.62 ± 0.73%. The higher recovery yield of TGF-β2 than of IGF-I represents the opposite situation compared to the results for serocolostrum presented in Section 2.2. This is due to different concentrations of the two GFs of interest in these dairy products. As noted earlier, in concentrated serocolostrum, IGF-I predominates over TGF-β2 almost two-fold (305.16 ± 2.34 ng/mL vs. 172.55 ± 2.41 ng/mL), while in acid whey tested, the situation is the opposite; in particular, the concentration of IGF-I and TGF-β2 was 6.22 ± 0.18 ng/mL and 11.67 ± 8.49 ng/mL, respectively, after a concentration of 5 kDa UF. Thereby, the concentration of GFs in serocolostrum before EDFM is more than 40 times higher for IGF-I and more than 10 times higher for TGF-β2 despite the high difference in the concentration factor achieved during UF (2.38× for serocolostrum and 16.3× for acid whey by volume). Thus, the significantly lower recovery yield values in the case of IGF-I for acid whey compared to serocolostrum (1.62 ± 0.73% vs. 5.53 ± 0.55% at pH 3.05, respectively) as well as the predominant migration of TGF-β2 from the feed to the recovery compartments confirm the conclusions made in Section 2.2 and also described in the articles of Aider et al. [30] and Bargeman et al. [31], where the transport rate increase with an increase in the concentration of substances in the feed solution during electromembrane filtration was reported.

**Figure 7:**
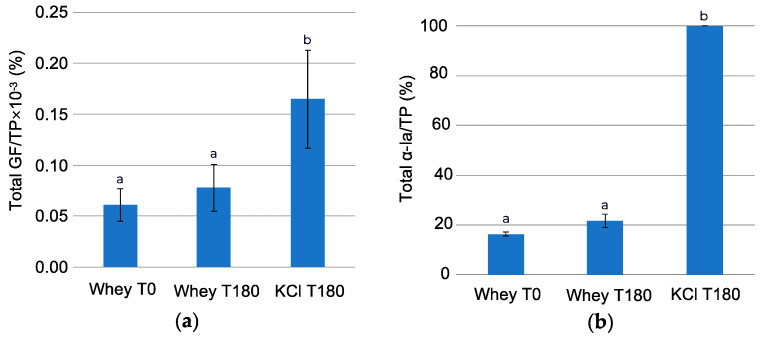
Enrichment in (**a**) the GFs of interest (IGF-I+TGF-β2) and (**b**) α-lactalbumin in the feed (at T0 and T180) and the recovery compartment at T180. Data with different letters are significantly different.

A protein content analysis with the Dumas method showed that the protein percentage was 41.93 ± 1.71% in the feed solution at T0, 41.05 ± 0.93% at T180, and 16.56 ± 3.38% on a dry basis in the recovery solution at T180. Based on these results and on the relative area under MS chromatograms, the enrichment ratio of total GFs by total protein as well as α-lactalbumin to total protein in the feed (at T0 and T180) and recovery solution (at T180) were calculated and presented in Figure 7.

The ratio of total GFs to total protein was 2.7× higher in the recovery solution than in the feed solution and 6.1× higher in the case of a ratio of α-lactalbumin to total protein. The significant difference in the ratio of total GFs to total protein between solutions in recovery and feed compartments in the case of acid whey and serocolostrum (2.7× vs. 60×, respectively) could be explained by their compositions. Indeed, it was found that protein composition in the recovery compartment solution during the EDFM of acid whey was higher than in the case of serocolostrum (247.19 ± 145.04 ug/mL vs. 89 ± 9 ug/mL at pH 3.05). The difference in protein concentration may be caused by the different concentration factor achieved during UF pretreatment of these dairy products. Indeed, acid whey was concentrated almost seven times more by volume than serocolostrum to achieve a higher GF content before EDFM. It should be noted that during the UF of dairy products using a 5 kDa membrane, proteins are retained due to the relatively large molecular size [39]. At the same time, as noted earlier, the concentration of the GFs of interest in acid whey was significantly lower than in serocolostrum initially [4]. These two aspects cause lower values of enrichment in GFs achieved for the case of acid whey.

Thus, these complimentary tests confirmed the previous results and that EDFM allowed for the production of GF-enriched fractions not only from serocolostrum but also from an acid whey solution.

## 3. Materials and Methods

### 3.1. Materials

Chemicals such as HCl and NaOH were bought from Fischer Scientific (Montreal, QC, Canada), and KCl and NaCl were obtained from BDH (VWR International Inc., Mississauga, ON, Canada). Na_2_SO_4_ was supplied by ACP Inc. (Montreal, QC, Canada).

Commercial food grade Neosepta ion-exchange membranes: anion-exchange membranes (AMX) and cation-exchange membranes (CMX) were bought from Astom Corp. (Tokyo, Japan). The ultrafiltration membrane, polyethersulfone (PES, commercial code: MQ) 300 kDa, was purchased from Synder Filtration Inc. (Vacaville, CA, USA). The thickness and conductivity of AMX, CMX, and PES membranes were performed according to the methods described by [28].

Colostrum was kindly provided by Centre de recherche en sciences animales de Deschambault (Quebec, QC, Canada). The choice of colostrum for the pretreatment optimization and feasibility demonstration was based on its relatively high GF content in comparison with other dairy solutions, such as milk and whey [4], making it a favorable choice to demonstrate the feasibility of the proposed process.

Quebon skim milk (Agropur Dairy Cooperative, Longueuil, QC, Canada) was used as an initial solution to produce an acid whey. This product was used in a second part of this study to demonstrate the potential use of this developed process for other dairy products since acid whey is a major concern for the dairy industry due to the volumes produced each year and its difficulty for further use, in contrast to sweet whey [40,41]. It is a by-product from acid-coagulated cheese (cottage cheese, ricotta cheese, etc.), Greek yogurt, and caseinate industries [38,42], which makes it a valuable source for GF recovery.

### 3.2. Pretreatment of Colostrum

Defatting of colostrum was carried out using Skimmer 100 L/h (DeLaval Canada, Peterborough, ON, Canada). Then, different acidification pretests (1 M HCl and 10% acetic acid as well as different dilution ratios of defatted colostrum, details are described in Section 2.2), followed by centrifugation using Avanti J-E high-speed centrifuge (Beckman Coulter Inc., Brea, CA, USA) for 20 min at 11,000× *g* at 4 °C were performed to remove caseins from defatted colostrum.

However, considering the very low concentration of GFs (<0.001 g/L in undiluted serocolostrum), obtained serocolostrum was thus concentrated (2.38× by volume) by ultrafiltration (UF) using GEA membrane filtration pilot plant (model L, Dusseldorf, Germany) with a 2.13 m^2^ spiral wound 5 kDa PES UF (Synder Filtration Inc., Vacaville, CA, USA). Indeed, concentrating of the solution obtained was necessary to achieve a significant concentration of GFs, especially in the recovery compartment after EDFM (higher than minimum detectable dose of ELISA kits (R&D system company, Minneapolis, MN, USA)). Obtained results suggested that IGF-I and TGF-β2 were concentrated 1.22× (305.16 ± 2.34 ng/mL) and 1.35× (172.55 ± 2.41 ng/mL), respectively, compared to their concentration in the raw serocolostrum (IGF-I: 252.07 ± 10.34 ng/mL; TGF-β2: 129.01 ± 7.74 ng/mL). Thus, a defatted and concentrated serocolostrum was obtained and used for EDFM experiments.

### 3.3. Preparation of Acid Whey

Skim milk was diluted in equal volumes with distilled water and then acidified with 1 M HCl until the pH value of 3.8 was reached, after which centrifugation under the same conditions as in the case of colostrum pretreatment was performed (20 min, 11,000× *g*, 4 °C). It should be noted that the concentration of GFs in whey is even lower than in milk and colostrum [4]. Indeed, TGF-β concentration in undiluted milk is 1.8 ± 0.3 ng/mL (vs. 3.7 ± 0.7 ng/mL in acidified whey) [43], while IGF-I concentration values vary widely in the literature. Thus, Daxenberger et al. [44] showed that IGF-I concentration in 5777 random milk samples ranges from 1.0 to 83 ng/mL. Thereby, prepared acid whey was concentrated through two repetitions to an averaged 16.3× by volume using the same filtration system as previously described in Section 3.2. ELISA results showed that TGF-β2 was concentrated around 3.5× compared to its initial concentration in acid whey (11.67 ± 8.49 ng/mL). Prepared concentrated acid whey was subjected to EDFM under the conditions indicated in Section 3.4, and at a pH of 3.05, 3 repetitions of EDFM were carried-out.

### 3.4. Electrodialysis Experiments

#### 3.4.1. ED Cell and EDFM Configuration

**Figure 8:**
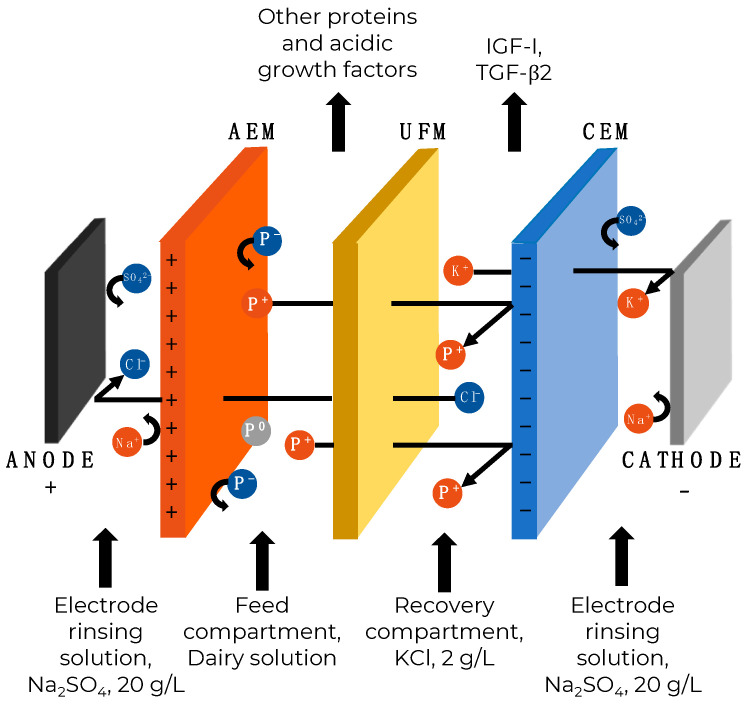
EDFM configuration, consisting of one recovery compartment, to produce GF-enriched fractions. AEM: Anion exchange membrane; UFM: Ultrafiltration membrane; CEM: Cation exchange membrane; P^+^: Positively charged protein; P^−^: Negatively charged protein; P^0^: Neutral protein.

MP-type ED cell manufactured by ElectroCell Systems AB company (Täby, Sweden) was used for all EDFM experiments. One PES 300 kDa ultrafiltration membrane was stacked in between AMX and CMX in the ED cell to obtain an EDFM configuration consisting of only one recovery (cationic) compartment (Figure 8). Such configuration was chosen following preliminary tests to allow for the migration of GFs of interest (IGF-I and TGF-β2) to the recovery compartment and the other proteins to remain in the feed compartment [14]. The effective surface area of filtration membrane used was 100 cm^2^. The EDFM configuration consisted of three recirculation compartments: one for cationic proteins and GF recovery, one for feed solution, and one for electrode rinsing solution (electrode rinsing loop was split into two circuits at the inlet of the cell). Recovery and feed compartment consisted of 500 mL of KCl (2 g/L) and 500 mL of feed solution, respectively, and electrode rinsing solution compartment contained 800 mL of Na_2_SO_4_ (20 g/L) solution. The solutions were circulated using three centrifugal pumps (Model WMD-30LFY-115, Iwaki Walchem Corporation, Holliston, MA, USA) and the flow rates were controlled at 500 mL/min for the feed and recovery compartments and 1000 mL/min for the electrode rinsing compartment using flow meters (Aalborg Instruments and Control Inc., Orangeburg, NY, USA).

#### 3.4.2. EDFM Protocol

Electroseparation of serocolostrum was performed in a batch process with a constant electric field strength of 2.9 V/cm (distance between two electrodes = 3.1 cm and a 9 V electrode potential difference) for a duration of 180 min according to [45]. The choice of the electrode potential difference is based on preliminary tests with the same experimental conditions. Thus, potential difference of 9 V corresponds to the 80% of the calculated limiting current density. EDFM was carried out at 4 different pH conditions: 4.25, 3.85, 3.45, and 3.05, chosen based on the preliminary results as well as physicochemical characteristics of the GFs (such as isoelectric points: 7.8–8.5 for IGF-I [4] and ≈7.7 for TGF-β2 [3,4]). For each pH condition, the conductivity of feed and recovery compartments were kept constant at their initial values by addition of KCl salt. To determine the migration kinetics of total proteins over the experimental period, 2 mL samples were collected from the feed and recovery compartments before applying voltage (0 min) as well as at 30, 60, 120, and 180 min. The samples collected at 180 min from recovery compartment were used to quantify the migrated proteins and GFs in that compartment with LC-MS and ELISA, respectively. After EDFM experiment, the cationic protein fractions and the feed solution recovered from their respective compartments were freeze dried and stored at 4 °C until analyzed further. Three independent EDFM experiments were carried out for each pH condition.

### 3.5. Performance Evaluation

#### 3.5.1. Total Protein Concentration and Migration Rate

Total protein concentrations in cationic recovery and feed compartments were determined using microBCA (µBCA) protein assay (Pierce, Rockford, IL, USA) from the samples withdrawn at the different time interval during EDFM (0, 30, 60, 120, and 180 min). The absorbance was read at 562 nm on a microplate reader (xMark, Bio-Rad, Hercules, CA, USA). Concentration was determined with a standard curve in a range of 0–40 µg/mL of bovine serum albumin (BSA) [28].

For all pH conditions, global rate of protein migration (g/m^2^·h) was calculated by dividing the total amount of proteins (g) migrated to the cationic recovery compartment at T = 180 min, determined with μBCA, by effective surface area of membrane (m^2^) and duration of EDFM process (h) (Equation (1)). High concentration of proteins in recovery compartment is associated with its high migration rate to that compartment.

(1) $$Migration\;rate\;(g/m^{2}\cdot h)=\frac{Total\;amount\;of\;protein\;in\;recovery\;compartment\;\left(g\right)}{Area\;\left(m^{2}\right)\;*\;Time\;(h)}$$

#### 3.5.2. Protein Characterization and Quantification

The samples recovered at the end of EDFM (T = 180 min) from the feed and the recovery compartments were analyzed with RP-UPLC. Samples were filtered through 0.22 µm PVDF syringe filter (Chromatographic Specialties Inc., Brockville, ON, Canada) into a glass vial. The sample was loaded (0.5 µL) onto an AdvanceBio RP-mAb Diphenyl column (2.1 × 100 mm, 3.5 micron, Agilent, Santa Clara, CA, USA). The column was operated at a flow rate of 400 µL/min at 60 °C. The gradient consisted of solvent A (LC-MS grade water with 0.1% formic acid) and solvent B (LC-MS grade ACN with 0.1% formic acid) starting at 30% B for 3 min, ramping to 40% B for 7 min, then 50% up to 9 min. It was then ramped to 90% B for 9.50 min, held for 10.50 min, then ramped back to initial conditions for 12 min.

A hybrid ion mobility quadrupole TOF mass spectrometer (6560 high-definition mass spectrometry (IM-Q-TOF), Agilent, Santa Clara, USA) was used to identify and quantify the relative abundances of proteins, such as α-la, β-lg, GMP, etc. All LC-MS/MS experiments were acquired using Q-TOF. Signals were recorded in positive mode at Extended Dynamic Range, 2 Ghz, 3200 *m*/*z* with a scan range between 100 and 3200 *m*/*z*. Nitrogen was used as the drying gas at 13.0 L/min and 150 °C and as nebulizer gas at 30 psig. The capillary voltage was set at 3500 V, the nozzle voltage at 300 V, and the fragmentor at 400 V. The instrument was calibrated using an ESI-L low concentration tuning mix (Agilent Technologies, Santa Clara, CA, USA). Data acquisition and analysis was achieved using the Agilent Mass Hunter Software package (LC/MS Data Acquisition, Version B.09.00; Qualitative Analysis for IM-MS, Version 10.0; and BioConfirm Software 10.0).

#### 3.5.3. Growth Factors Quantification, Recovery Yield, and Enrichment

To quantify GFs of interest (IGF-I and TGF-β2), sandwich Enzyme-Linked ImmunoSorbent Assay (ELISA) tests were carried out. The commercial kit used was Human IGF-I Quantikine (DG100B) and Human TGF-β2 Quantikine (DB 250) ELISA kit since the sequences of both GFs of human are identical to that of bovine [46,47]. The tests were performed according to the manufacturer’s recommendations. The concentration of GFs was expressed in ng/mL.

For the quantification of IGF-I, standards and samples were pipetted in triplicate into the wells of the provided microplate, covered with adhesive strip, and incubated with agitation. The microplate used was precoated with a monoclonal antibody specific for human IGF-I so that any IGF-I present in standards and samples would bind to it. Then, a washing step was carried out to remove the unbound substances. Next, an enzyme-linked monoclonal antibody specific for human IGF-I was added to the wells followed by washing to remove any unbound antibody–enzyme reagent. Then, a substrate solution was added to the wells and incubated with agitation protected from light. Color development in this step is proportional to the amount of IGF-I bounded in the initial step. Finally, the reaction/color development was stopped by adding stop solution (2 N sulfuric acid), and the absorbance of samples was read at 450 nm and 540 nm on a microplate reader (xMark, Bio-Rad, Hercules, CA, USA). Finally, the absorbance at 540 nm was subtracted from the absorbance at 450 nm to correct the optical imperfections in the plate.

For TGF-β2, prior to quantification by sandwich ELISA, its activation was carried out since more than 90% of its total concentration in colostrum is present in an inactive form and can be activated by changing ionic strength, by acidification, or by proteolytic enzymes [48]. Briefly, the samples (125 μL) were activated to immunoreactive form by acidification: by adding 25 μL of 1 N HCl followed by incubation for 10 min. Afterward, the samples were neutralized by adding 25 μL of 1.2 N NaOH/0.5 M HEPES buffer. Finally, similar steps as those for IGF-I were assessed to quantify the activated TGF-β2 in the samples.

The recovery yield of GFs was calculated using the following equation, Equation (2):

(2) $$Recovery\;yield=\frac{Total\;amount\;of\;growth\;factor\;in\;recovery\;compartment\;(ng)}{Total\;amount\;of\;growth\;factor\;in\;feed\;solution\;(ng)}\times 100$$

To determine if there is an enrichment of GFs in the recovery compartment, the following equation, Equation (3), was used:

(3) $$Enrichment\;of\;growth\;factors\;\left(\%\right)=\frac{Total\;amount\;of\;growth\;factors\;in\;a\;compartment\;\left(ng\right)}{Total\;protein\;in\;the\;same\;compartment\;\left(ng\right)}\times 100$$

#### 3.5.4. Energy Consumption

##### Total Energy Consumption

The energy consumption of each ED experiment for each membrane type was calculated to measure their electrical efficiency using the following equation, Equation (4) [37]:

(4) $$EC=U\int_{0}^{t}I\left(t\right)dt,$$

where EC is the energy consumption (in Wh), I is the intensity (in A), U is the voltage (in V), and t is the duration of the treatment (in h).

##### Relative Energy Consumption for Growth Factors

The relative energy consumption in Wh/μg of GFs during EDFM treatment for GFs of interest (IGF-I+TGF-β2) was calculated by dividing the total energy consumed (Wh) by the total amount of GFs (μg) obtained at the end of the process using the following equation, Equation (5):

(5) $$Relative\;energy\;consumption\;(Wh/\mu g)=\frac{Total\;energy\;(Wh)}{Total\;amount\;of\;growth\;factors\;(\mu g)}$$

#### 3.5.5. Statistical Analyses

All experiments on separation of GFs from serocolostrum and acid whey were performed in triplicates. The data were subjected to one-way analyses of variance (ANOVA) and Tukey tests at a probability level of 0.05 using SigmaPlot software (Version 14.0, Systat Software, San Jose, CA, USA).

## 4. Conclusions

This work demonstrates, for the first time, the feasibility of EDFM technology to produce GF-enriched fractions from dairy solutions. Based on the pretreatment results, it was demonstrated that the 1:1 dilution of defatted colostrum with deionized water to decrease mineral content followed by the preconcentration of GFs by UF is necessary to increase the concentration of the latter at a certain level and allow for these compounds to migrate to the recovery compartment during EDFM. An important impact of the pH of the solution on the migration of GFs and, therefore, the recovery yield during EDFM as well as the migration of other proteins was noticed. However, regardless of pH values, the recovered fractions were significantly enriched, up to 60-fold in the case of serocolostrum and up to 2.7-fold for acid whey at pH 3.05, with the GFs of interest compared to that of the feed solution. Indeed, the highest migration of GFs was achieved at the lowest pH condition used due to the synergistic effect of different phenomena: the increased electrophoretic mobility of the GFs of interest due to the increase in their global net charge and electrostatic interaction (attraction) between charged GFs and membrane surface, thereby contributing to the facilitated migration through FM. Among four major proteins (GMPs, BSA, α-la, and β-lg) identified in the feed, only α-la and β-lg migrated in the recovery compartment during the EDFM of colostrum and only α-la in the case of whey.

Thus, this work suggested the potential application of EDFM technology to produce GF-enriched fractions in dairy industries. It could be concluded that in general, the proposed technology is a promising way for valorizing liquid waste from the dairy industry to extract valuable compounds such as GFs. However, further scaling-up experiments at the pilot scale with different dairy solutions and after their concentration should be realized. Thus, in the case of pilot and production scales, the energy consumptions must be recalculated vs. product quality/functionality and selling cost to confirm the application of such technology for GF separation and purification.

In addition, it should be mentioned that the main objective of this study was mainly a proof of concept, and the further optimization of the process parameters and conditions is necessary to obtain more efficient results in terms of GF enrichment and current efficiency. Moreover, the recovered stream as well as the feed may be subject to consequent treatment to obtain more valuable compounds for a reasonable business case. In addition, comparing EDFM with chromatography and conventional pressure-driven membranes, some benefits can still be obtained with respect to cost vs. protein/peptides selectivity. The sustainability issue is also an important factor in the industry dealing with new technologies. As ED and EDFM are mainly based on electricity, it is counted as a “green energy” [21], which is worth for further investigation.

## 5. Patents

The patent “Method of preparing a dairy fraction enriched in growth factor and said product” is based on the work reported in this manuscript (No. EP24160962.7, 1 March 2024).

## Figures and Tables

**Table 1:** The recovery yield of GFs of interest in the recovery compartment after 180 min of EDFM in the different conditions of pH.

pH Condition	IGF-I (%)	TGF-β2 (%)
3.05	5.53 ± 0.55 ^a^	1.50 ± 0.25 ^a^
3.45	4.42 ± 0.42 ^b^	2.00 ± 0.70 ^a^
3.85	1.44 ± 0.15 ^c^	0.27 ± 0.08 ^b^
4.25	0.83 ± 0.02 ^c^	0.20 ± 0.05 ^b^

## Data Availability

The data presented in this study are available on request from the corresponding author. The data are not publicly available due to ongoing industrial collaborative projects.
